# Accurate and sensitive TaqMan real-time PCR assays to detect and quantify simian malaria parasites *Plasmodium cynomolgi, Plasmodium inui* and *Plasmodium coatneyi*

**DOI:** 10.1186/s12936-026-05955-4

**Published:** 2026-06-08

**Authors:** Muhammad Mirza Ariffin, Khairunnisa Syaripuddin, Khamisah Abdul Kadir, David Lubim, Khatijah Yaman, Paul Cliff Simon Divis

**Affiliations:** 1 Malaria Research Centre, Faculty of Medicine & Health Sciences, Universiti Malaysia Sarawak, Kota Samarahan, Sarawak Malaysia; 2 Entomology and Pest Unit, Kapit Divisional Health Office, Kapit, Sarawak Malaysia; 3 Entomology and Pest Section, Sarawak State Health Department, Kuching, Sarawak Malaysia

**Keywords:** Malaria, *Plasmodiumcynomolgi*, *Plasmodiuminui*, *Plasmodiumcoatneyi*, Real-time PCR, TaqMan probe, Zoonosis

## Abstract

**Background:**

Human infections with simian parasites such as *Plasmodium inui, P. cynomolgi*, and *P. coatneyi* have been reported in several countries within Southeast Asia. Though infections caused by these parasites are mostly asymptomatic and mild, this could hamper the efforts to eradicate malaria in this region. In this report, we outlined the development and optimisation of new TaqMan-based real-time PCR assays to accurately identify and quantify *P. inui*, *P. cynomolgi*, and *P. coatneyi*.

**Methods:**

Species-specific hydrolysis probes were designed for real-time PCR assays targeting variable regions within the 18S rRNA genes of *P. inui, P. cynomolgi* and *P. coatneyi*. The assays were tested against plasmid DNA containing the target 18S rRNA gene as well as plasmid DNA harbouring 18S rRNA gene fragments of other human and simian *Plasmodium* species. The assays were further tested using genomic DNA samples from macaques and *Anopheles* mosquitoes that were previously confirmed to be positive for simian *Plasmodium* by nested PCR assays.

**Results:**

The developed assays demonstrated high specificity, amplifying only the target 18S rRNA gene of their respective *Plasmodium* species. The assays were also able to detect and quantify as few as 10 copies of template DNA per PCR reaction, which is equivalent to approximately five parasites, with amplification efficiencies of > 95%. The generated standard curves for each assay showed linear relationship between the copy numbers (10–10^6^ copies) and the cycle threshold (Ct) values. The accuracy of the assays was further supported by their ability to produce results equivalent to those obtained using conventional nested PCR assays.

**Conclusions:**

The newly developed assays can accurately detect and quantify *P. inui, P. cynomolgi* and *P. coatneyi*, with limit of detection of approximately five parasites. Given the streamlined nature of real-time PCR, these assays could be implemented for routine malaria surveillance in areas where zoonotic malaria is prevalent. In addition, these assays are suitable for the detection of submicroscopic infections and in mosquito samples, which often have low parasite densities.

**Supplementary Information:**

The online version contains supplementary material available at 10.1186/s12936-026-05955-4.

## Background

Malaria is a parasitic life-threatening febrile illness caused by the protozoa of the genus *Plasmodium* and is transmitted through the bites of female *Anopheles* mosquitoes [1, 2]. Though successfully eradicated in many parts of the world, it remains endemic in several countries within the Southeast Asia region. In Malaysia for instance, indigenous malaria infections caused by the four human *Plasmodium* parasites (*P. falciparum, P. vivax, P. ovale,* and *P. malariae*) are no longer considered a threat to the public health. Despite this, zoonotic infections with the simian malaria parasites, primarily *P. knowlesi*, are increasing exponentially in the recent years [3–6].

Natural human infections with the simian *Plasmodium* parasites was initially regarded to be rare and was only deemed transmissible in laboratory-controlled settings [7]. However, in 2004, molecular assays were utilised to examine the blood samples from malaria patients that were positive for *P. malariae* through microscopic analysis, from Kapit Division of Sarawak, Malaysia [8]. Of the 208 samples subjected to nested PCR assays, none were positive for *P. malariae*, whereas more than 50% were found to be *P. knowlesi*-positive. Subsequent to this finding, several more countries across Southeast Asia began to report malaria cases in humans involving this particular simian parasite [9–12].

Apart from *P. knowlesi*, several other simian malaria parasites, including *P. cynomolgi*, *P. inui*, *P. eylesi*, *P. brasilianum*, *P. schwetzi*, and *P. simium*, have been reported to be transmissible to humans [1, 13, 14]. Since 2014, there has been an increasing number of natural *P. cynomolgi* infections reported among asymptomatic malaria patients within Southeast Asia, signifying the possibilities of zoonotic emergence by this simian parasite [15, 16]. Meanwhile, *P. inui*, *P. coatneyi,* and other simian *Plasmodium* parasites, were detected through molecular screening of archived blood samples collected from the indigenous forest-fringe dwellers in Malaysia and among the febrile patients from malaria-endemic provinces of Thailand [15, 17]. These parasites have been described to naturally infect *Macaca nemestrina* (pig-tailed macaque), *Macaca fascicularis* (long-tailed macaque), and several other non-human primate species that are widespread throughout the Southeast Asian region [1, 18–20].

The morphologies of several simian *Plasmodium* parasites are reported to be highly similar when observed microscopically [21]. *P. cynomolgi* for instance is reported to be morphologically identical to *P. vivax*, while *P. inui* and *P. coatneyi* highly resemble *P. malariae* and *P. falciparum*, respectively [1, 15]. Due to this morphological similarity, accurate species identification using conventional microscopy technique remains challenging [22, 23]. However, given its lower cost and wide availability, microscopy is still routinely used in many diagnostic laboratories, particularly in resource-limited settings [24, 25].

To overcome the limitations of microscopy, molecular assays such as nested PCR were developed to improve the sensitivity and accuracy of *Plasmodium* species detection and identification [8, 26, 27]. However, recent studies have highlighted that conventional nested PCR assays may occasionally fail to identify the *Plasmodium* parasites in *Anopheles* mosquitoes due to the very low copy number of the parasites [28, 29].

In addition to nested PCR, quantitative PCR (qPCR) assays have been developed to enable simultaneous detection and quantification of *Plasmodium* DNA [30–32]. However, most existing qPCR assays focus primarily on human malaria parasites and *P. knowlesi*, whereas assays targeting other simian *Plasmodium* species that infect humans remain limited. To address this gap, this study describes the development and optimisation of new real-time PCR assays employing species-specific TaqMan probes for *P. cynomolgi, P. inui,* and *P. coatneyi*.

## Methods

### Preparation of plasmid DNA samples from glycerol stocks

Three recombinant plasmids, each containing the A-type 18S rRNA gene insert of *P. inui, P. coatneyi*, or *P. cynomolgi*, were prepared by reviving archived *E. coli* glycerol stocks prepared in previous study by Lee et al. [20] (Additional File 1). Plasmid DNA was extracted from overnight cultures using a commercial miniprep kit (PureLink™ Quick Plasmid Miniprep Kit, Invitrogen, USA) according to the manufacturer’s instructions. The concentration and purity of the extracted plasmids were measured using the Nanodrop One spectrophotometer (Thermo Fisher Scientific, USA) and are provided in Additional File 1.

### Development of species-specific TaqMan probes

The developed qPCR assays utilised the TaqMan probe-based chemistry to detect the target DNA. Species-specific probes for *P. inui, P. cynomolgi*, and *P. coatneyi* were designed from the alignment of 40 reference 18S rRNA gene sequences retrieved from GenBank, representing these target species as well as other simian and human *Plasmodium* species (*P. knowlesi, P. fieldi, P. simiovale, P. falciparum, P. vivax, P. malariae*, and *P. ovale*) (Additional File 2). The dataset included both A-type and S-type 18S rRNA gene isoforms derived from different hosts, including non-human primates, humans, and mosquito vectors.

The alignment was generated using the Clustal W algorithm in the Unipro UGENE software (version 48.0) and the specific binding site for each probe was manually determined. The 5’ and 3’ ends of the probes were labelled with the 6-carboxyfluorescein (FAM) fluorophore and Iowa Black™ fluorescent quencher (FQ), respectively, together with an internal ZEN quencher, forming a double-quenched probe design [33]. These probes were subsequently synthesised by Integrated DNA Technologies (Singapore).

### qPCR reaction setup and cycling conditions

The qPCR assays were performed by combining the newly developed TaqMan probes with previously published *Plasmodium*-specific PCR primers, Plasmo1 (5’-GTT AAG GGA GTG AAG ACG ATC AGA-3’) and Plasmo2 (5’-AAC CCA AAG ACT TTG ATT TCT CAT AA-3’) [34]. A singleplex qPCR format was employed, in which each reaction contained only one species-specific probe to allow independent amplification and quantification of *P. inui, P. coatneyi*, and *P. cynomolgi*.

The assays were performed in a 25 μL reaction mixture comprising 5 μL of DNA template, 12.5 μL of TaqMan Universal Mastermix (Applied Biosystems, USA), 80 nM of TaqMan probe and 200 nM of each PCR primer in the LightCycler^®^ 480 Instrument II system (Roche Diagnostics, USA). The reaction conditions used were as follows: 2 min incubation at 50 °C, initial denaturation at 95 °C for 10 min, followed by 45 cycles of denaturation at 95 °C for 15 s and annealing/extension at 60 °C for 1 min. The fluorescence data was acquired during the annealing/extension step and the cycle threshold (C_*t*_) values were obtained by adjusting the threshold 10 times the standard deviation above the background fluorescence baseline.

### Specificity and sensitivity assessment of the developed qPCR assays

To rule out potential cross-reactivity with other *Plasmodium* species, each assay was tested against molecularly-confirmed archived plasmid DNA samples containing the inserts of asexually or sexually-transcribed 18S rRNA gene of *P. vivax, P. knowlesi, P. fieldi, P. inui, P. cynomolgi, P. coatneyi* and *P. simiovale* from previous study [20]. In addition, each assay was tested against genomic DNA from three blood samples of *P. falciparum*, *P. vivax*, *P. ovale*, and *P. malariae* obtained from patients admitted at Kapit Hospital [35, 36]. *Plasmodium* species in these samples were identified and confirmed by nested PCR assays (Additional File 3).

The assays were further tested using archived genomic DNA samples from ten macaques that tested positive for simian *Plasmodium* parasites by nested PCR assays performed in a previous study [20] (refer to Table 4). Two of the macaques were positive for *P. inui*, while the remaining four were positive for *P. coatneyi* and *P. cynomolgi*, respectively. In addition, we received 607 dried *Anopheles* mosquito samples from the entomologist at the Kapit Divisional Health Office, Sarawak, during the routine vector control surveillance for malaria from 2020 to 2022. All mosquitoes were morphologically identified at the time of collection. Of these, 62.1% were identified as *An. donaldi*, followed by *An. latens* (17.0%), *An. tessellatus* (10.5%), *An. maculatus* (6.3%), *An. barbirostris* (3.1%), *An. barbumbrosus* (0.7%), *An. macarthuri* (0.2%), and *An. sundaicus* (0.2%). Genomic DNA was extracted from the mosquitoes using the DNeasy Blood and Tissue Kit (QIAGEN, Germany) according to the manufacturer’s protocol. Subsequently, nested PCR assays described by Lee et al. [20] were performed to detect the presence of *Plasmodium* DNA in the mosquitoes.

To determine the limit of detection (LOD) for each qPCR assay, the extracted plasmid DNA containing the A-type 18S rRNA gene of *P. inui, P. coatneyi* or *P. cynomolgi* was serially diluted in nuclease-free water to generate working solutions with concentrations ranging from 10^6^ to 1 copies of plasmid DNA per μL, according to the protocol by Applied Biosystems [37]. Each dilution was analysed in triplicate to ensure reproducibility. The LOD was defined as the lowest concentration of target DNA that was consistently detected across replicates. Subsequently, standard curves for each assay were generated by plotting the mean Ct values against the logarithm of the plasmid DNA copy number. In addition, the efficiency of each assay was calculated from the slope of the standard curve using the formula: Efficiency (%) = (10^−1/slope^–1) × 100.

## Results

### Species-specific TaqMan probes for *P. inui, P. coatneyi*, and *P. cynomolgi*

**Fig. 1:**
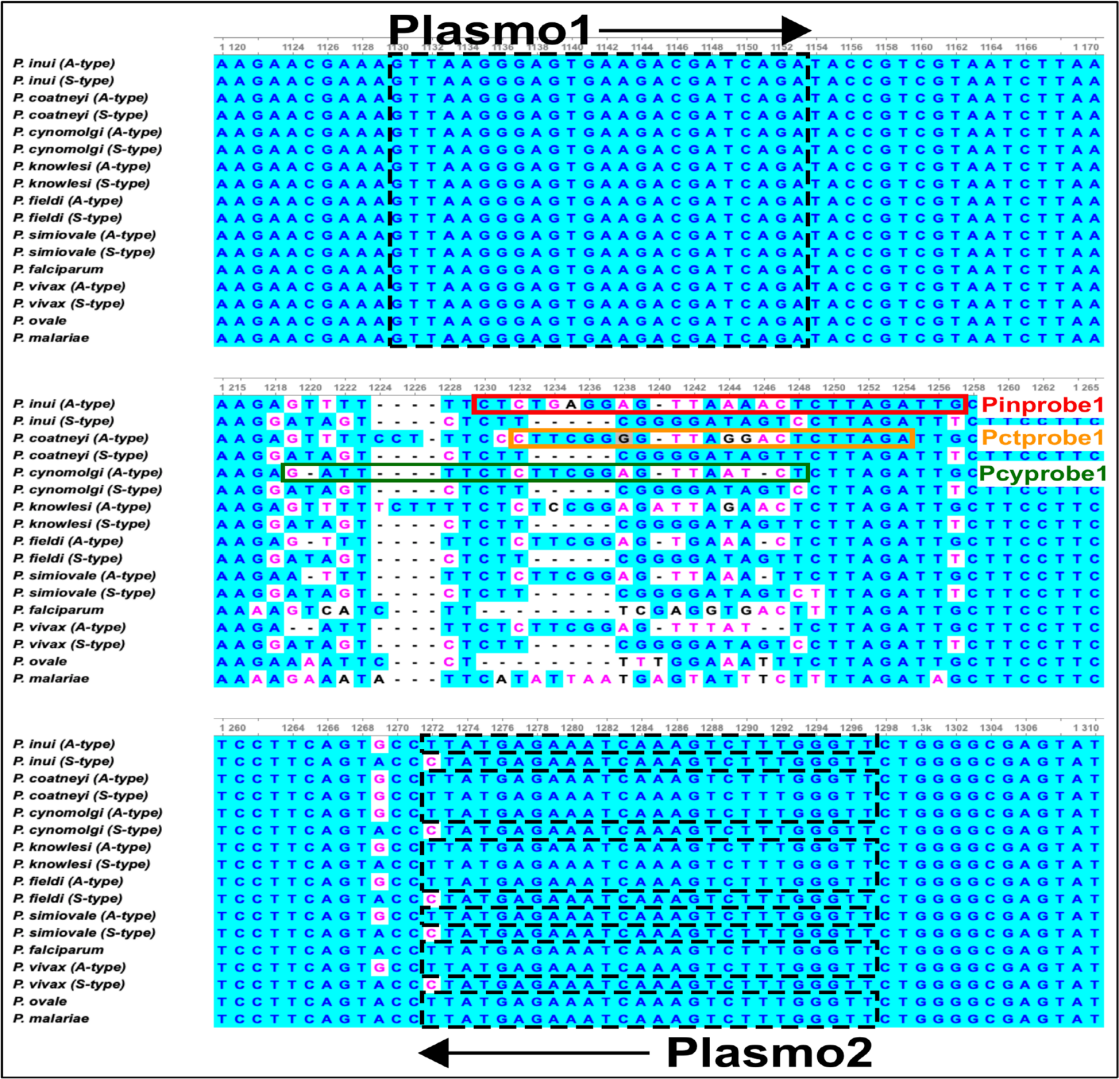
Location of PCR primers Plasmo1 and Plasmo 2, and the newly developed probe for *P. inui* (Pinprobe1), *P. coatneyi* (Pctprobe1) and *P. cynomolgi* (Pcyprobe1) within the aligned *Plasmodium* 18S rRNA gene consensus sequences. The consensus sequence for each *Plasmodium* species was generated by aligning the reference sequences of the 18S rRNA gene retrieved from GenBank. The accession numbers of the reference sequences used are provided in Additional File 2

Preliminary BLAST searches confirmed that each probe sequence was specific to its respective *Plasmodium* species and showed no similarity to human or other pathogen DNA. The nucleotide sequences and melting temperatures (*T*_*m*_) of each probe are provided in Table 1. The positions of the probes within the aligned *Plasmodium* SSU rRNA gene sequences are shown in Fig. 1.

**Table 1:** The nucleotide sequence and melting temperature of the newly developed TaqMan probes for *P. inui, P. coatneyi,* and *P. cynomolgi*

Code	*Plasmodium* species	Strand	Sequence (5ʹ–3ʹ)	*T*_*m*_ (°C)
Pinprobe1	*P. inui*	Reverse	CAA TCT AAG AGT TTT AAC TCC TCA GAG	55.2
Pctprobe1	*P. coatneyi*	Reverse	TCT AAG AGT CCT AAC CCC GAA G	54.8
Pcyprobe1	*P. cynomolgi*	Reverse	AG ATT AAC TCC GAA GAG AAA ATC	50.3

Each probe was labelled with FAM fluorophore and Iowa Black™ FQ quencher at the 5ʹ and 3ʹ ends, respectively, along with another ZEN internal quencher

### Specificity of qPCR assays

When each probe was tested against plasmids containing the A- or S-type 18S rRNA from several *Plasmodium* species, no cross-reactivity against the S-type 18S rRNA genes and other closely related *Plasmodium* species were observed (Table 2). Assay specificity was affirmed when tested against archived genomic DNA samples from macaques with single simian *Plasmodium* infections confirmed by PCR assays in a previous study [20] (Table 3). Macaque samples positive for *P. inui* were detected exclusively by the *P. inui*-specific qPCR assay. Similarly, macaques with *P. coatneyi* and *P. cynomolgi* infections were detected only by their respective species-specific qPCR assays, with no amplification observed in non-target qPCR assays. When tested on four human *Plasmodium* species obtained from malaria patients admitted to Kapit Hospital, Sarawak from our previous studies, these qPCR assays showed no cross-reactivity, thereby confirming the specificity of the assays (Additional File 3).

**Table 2:** Specificity of the qPCR assays of *P. inui*, *P. coatneyi* and *P. cynomolgi* using plasmid DNA samples containing A- and S-type 18 rRNA genes of closely related *Plasmodium* species

*Plasmodium* species	18S rRNA gene type	Species-specific qPCR assays		
		*P. inui* C*t*	*P. coatneyi* C*t*	*P. cynomolgi* C*t*
*P. vivax*	A	n.d	n.d	n.d
	S			
*P. inui*	A	15.8	n.d	n.d
	S	n.d		
*P. coatneyi*	A	n.d	13.5	n.d
	S		n.d	
*P. cynomolgi*	A	n.d	n.d	18.8
	S			n.d
*P. knowlesi*	A	n.d	n.d	n.d
	S			
*P. fieldi*	A	n.d	n.d	n.d
*P. simiovale*	A	n.d	n.d	n.d

*Ct*: cycle threshold value representing the PCR cycle at which fluorescence exceeds the detection threshold. Lower C*t* values indicate higher initial template DNA concentration. Amplification was observed only for the target species with the A-type 18S rRNA gene, while no amplification was detected for S-type isoforms or non- target species. *n.d.*: not detected (no amplification observed)

**Table 3:** Detection of *P. inui, P. coatneyi*, and *P. cynomolgi* in genomic DNA samples from macaques (*Macaca sp.*) and *Anopheles* mosquitoes obtained from Kapit Division, Sarawak

Samples	Code	Hosts	*Plasmodium* species identified by nested PCR assay	qPCR assays		
				*P. inui*	*P. coatneyi*	*P. cynomolgi*
				C*t*	C*t*	C*t*
Macaques	LT45	*M. fascicularis*	*P. inui*	16.97	n.d	n.d
	LT113	*M. fascicularis*	*P. inui*	23.29	n.d	n.d
	KP-LT23	*M. fascicularis*	*P. coatneyi*	n.d	20.05	n.d
	KP-LT37	*M. fascicularis*	*P. coatneyi*	n.d	21.78	n.d
	LT68	*M. fascicularis*	*P. coatneyi*	n.d	21.43	n.d
	LT93	*M. fascicularis*	*P. coatneyi*	n.d	25.83	n.d
	KP-LT38	*M. fascicularis*	*P. cynomolgi*	n.d	n.d	34.30
	LT43R	*M. fascicularis*	*P. cynomolgi*	n.d	n.d	26.47
	AS01	*M. nemestrina*	*P. cynomolgi*	n.d	n.d	29.64
	LB-PT031	*M. nemestrina*	*P. cynomolgi*	n.d	n.d	27.09
*Anopheles*	DSB010	*An. donaldi*	*P. inui*	34.6	n.d	n.d
	TX017	*An. tesselatus*	*P. inui*	30.8	n.d	n.d
	RP004	*An. latens*	*P. coatneyi*	n.d	32.8	n.d
	TX254	*An. tesselatus*	*P. coatneyi*	n.d	26.8	n.d
	TX113	*An. donaldi*	*P. cynomolgi*	n.d	n.d	34.9
	TX203	*An. donaldi*	*P. cynomolgi*	n.d	n.d	28.2

*Ct*: cycle threshold value representing the PCR cycle at which fluorescence exceeds the detection threshold. Lower C*t* values indicate higher initial template DNA concentration. Only samples positive by the corresponding species-specific qPCR assay yielded C*t* values. *n.d.*: not detected (no amplification observed). The *Plasmodium* species in each macaque sample was confirmed by nested PCR assays performed in a previous study by Lee et al. [20]. The *Anopheles* mosquitoes were identified morphologically by the entomologist at the Kapit Divisional Health Office, Sarawak, at the time of collection. All mosquitoes received were dried, which prevented the dissection of the salivary glands for sporozoite detection.

### Detection of *P. inui, P. coatneyi, *and* P. cynomolgi* in genomic DNA of *Anopheles* mosquitoes

The 607 whole *Anopheles* mosquito samples received from the Kapit Divisional Health Office were dried, therefore, examination of salivary glands for plasmodial sporozoites was not performed. Nested PCR assays indicated that six mosquitoes were positive for *Plasmodium* DNA. When tested with the newly developed qPCR assays, these mosquitoes showed single infections either *P. inui, P. coatneyi* or P*. cynomolgi*, consistent with the nested PCR findings (Table 3). The remaining mosquitoes tested negative for *Plasmodium* species by the qPCR assays.

### Limit of detection of qPCR assay

**Fig. 2:**
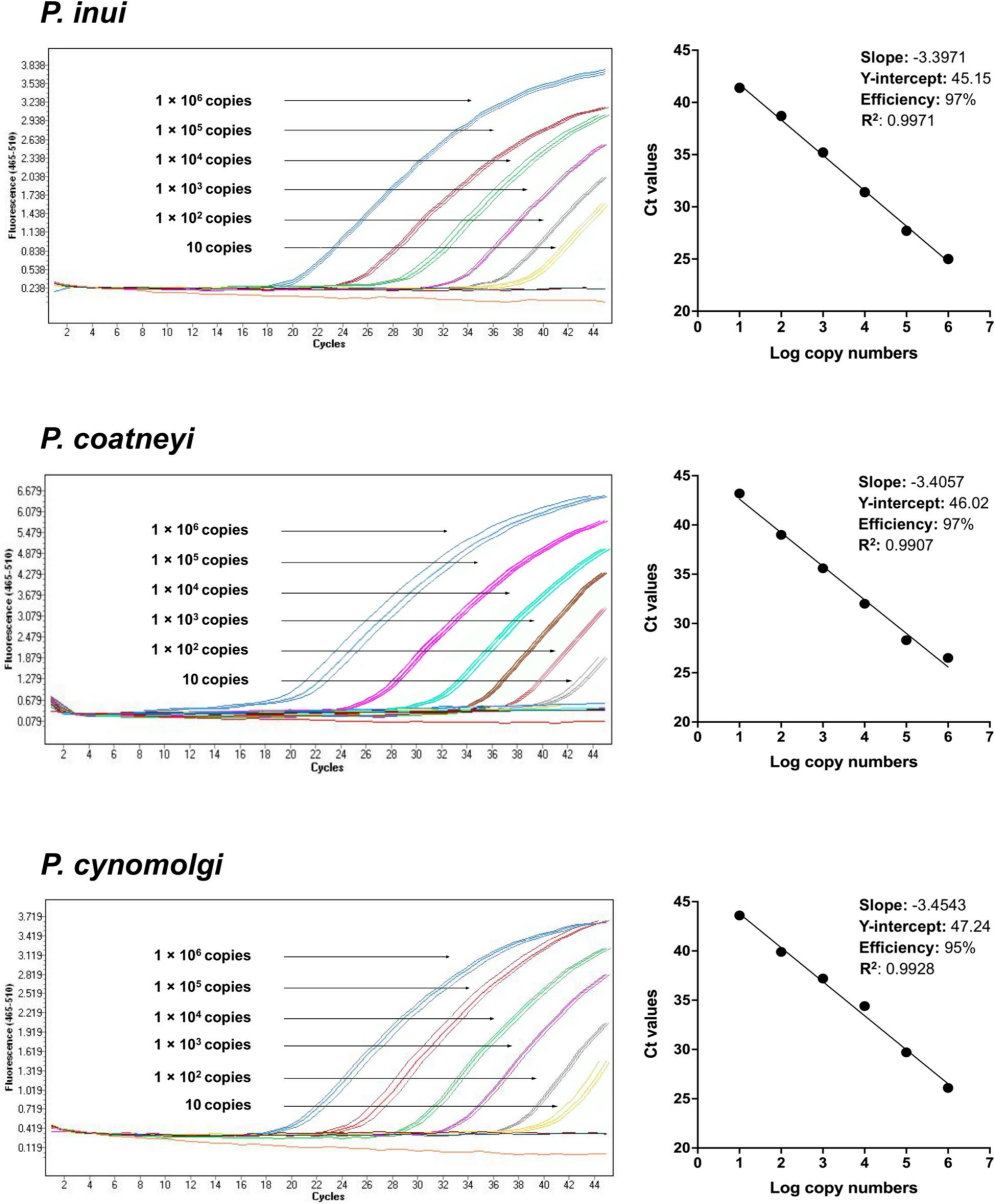
The qPCR amplification curves generated when testing each assay against tenfold serial dilutions of plasmid DNA containing the target SSU rRNA gene for *P. inui*, *P. coatneyi*, and *P. cynomolgi*. All assays achieved a limit of detection of 10 copies of target DNA per reaction. Standard curves generated for each assay, shown in the right panel, illustrate the linear relationship between the average C*t* value recorded for each dilution and logarithm of plasmid DNA copy numbers. The calculated slopes, amplification efficiencies, and correlation coefficients (R^2^) indicate the robustness, efficiency, and reproducibility of the assays

To test the sensitivity of the qPCR assays, each assay demonstrated a detection limit of 10 copies of the target 18S rRNA gene per reaction (Fig. 2, Additional file 4). Standard curves constructed by plotting C*t* values against the logarithm of plasmid DNA copy number indicated strong linearity (R^2^ > 0.99) for the *P. inui, P. cynomolgi*, and *P. coatneyi* assays (Fig. 2). Based on the slopes of the standard curves, the amplification efficiencies calculated for both *P. inui* and *P. coatneyi* assays were 97%, whereas the *P. cynomolgi* assay exhibited slightly lower efficiency of 95%.

### Target copy number analysis and estimation of parasite detection limits

To estimate the limit of parasite detection by the qPCR assays, an in silico PCR analysis, which simulates primer–probe binding and amplification using Basic Local Alignment Search Tool (BLAST) analysis, was performed against the multi-copies of the A-type of 18S rRNA genes in the respective genome sequences obtained from GenBank. PCR primers Plasmo1 and Plasmo2 bind against the A-type of 18S rRNA gene at four chromosomes within the *P. cynomolgi* strain B genome (Table 4). However, Pcyprobe1 was shown to bind against the A-type gene located at two of these chromosomes, which were chromosomes 3 and 10. A similar pattern was observed for *P. coatneyi* strain Hackeri genome, where two copies of the Pctprobe1 target sites were identified in different chromosomes in the genome. Given that both *P. cynomolgi* and *P. coatneyi* assays had a limit of detection (LOD) of 10 copies/µL, the minimum detectable parasite load was estimated to be approximately five parasites for each species.

**Table 4:** Mapping of *Plasmodium* genus-specific primers (Plasmo1 and Plasmo2) and the species-specific qPCR probes across selected *Plasmodium* genomes

*Plasmodium* species (strain)	Chr	Accession number	Plasmo1 and Plasmo2 primers	Pcyprobe1	Pctprobe1	Pinprobe1
*P. cynomolgi* (B)	3	NC020397	✓	✓	n.a	n.a
	5	NC020398	✓	×	n.a	n.a
	6	NC020399	✓	×	n.a	n.a
	10	NC020403	✓	✓	n.a	n.a
*P. coatneyi* (Hackeri)	3	NC033558	✓	n.a	✓	n.a
	5	NC033560	✓	n.a	×	n.a
	10	NC033565	✓	n.a	✓	n.a
*P. inui* (San Antonio)	Not assigned	NW008481872	✓	n.a	n.a	✔

The *Plasmodium* strains used and their corresponding chromosome (Chr.) information and accession numbers were obtained from the GenBank database. The mapping results represent the binding of each PCR primer and qPCR probe to the respective target sequences. A tick (✓) indicates successful binding to the target region, while a cross (×) indicates no detectable binding. “n.a” denotes that the probe is not applicable to the respective *Plasmodium* species, as it was not designed to target that species

In contrast, for *P. inui* strain San Antonio, the genomic sequence was not assigned to specific chromosomes in the GenBank database. Although the Pinprobe1 binding site was identified at one locus within the genome, the target copy number could not be determined. However, based on the multi-copy nature of the A-type 18S rRNA genes in the genomes of *P. cynomolgi* and *P. coatneyi*, the minimum detectable number of parasites is estimated to be within a similar range (approximately five parasites).

### Discussions

These newly-developed qPCR assays to detect of *P. inui, P. coatneyi* and *P. cynomolgi* served as an extension to the previously published assays by that detect the four human *Plasmodium* parasites and *P. knowlesi* [31, 34] Similar to both previous studies, these assays utilised *Plasmodium*-genus specific PCR primers that bind to the highly conserved region within the 18S rRNA gene of the wide range of *Plasmodium* species. These PCR primers complement with the newly designed *P. inui*-, *P. coatneyi*- and *P. cynomolgi*-specific probes that bind specifically to the variable sites within the 18S rRNA gene.

The high specificity observed in these qPCR assays is likely attributable to the target-specific characteristics of the Taqman probes. In contrast to SYBR Green-based qPCR, which detects fluorescence from double-stranded DNA products including non-specific amplicons, TaqMan assays generate fluorescence only when the probe hybridises to the correct target sequence during amplification [38, 39]. This probe-dependent detection reduces non-specific signal generation and lowers the risk of false-positive amplification, thereby enhancing the overall accuracy and reliability of the assay. In addition, SYBR Green-based assays typically require supplementary post-amplification procedures, such as melting curve analysis, to confirm the specificity of the amplified products [39].

In addition to the high specificity, these qPCR assays exhibited strong analytical performance, with their ability to detect as few as 10 copies of the A-type of 18S rRNA gene per reaction. Given that this gene is present in multiple copies within the *Plasmodium* genome, with copy numbers varying between *Plasmodium* species, this detection threshold (10 copies) corresponds to approximately five parasites per reaction [40–42]. This level of sensitivity is particularly important for detecting low-density infections, where parasite burdens are often low and may be missed by less sensitive detection methods, including microscopy [40, 43].

Although the species-specific qPCR assays in this study were developed based on well-established TaqMan principles, their key contribution lies in their application to target simian *Plasmodium* species that remain under-recognised in malaria surveillance. Current molecular diagnostic and surveillance efforts in zoonotic malaria-endemic regions are predominantly focused on *P. knowlesi*, potentially leading to under-detection or misidentification of other simian malaria parasites such as *P. cynomolgi*, *P. inui*, and *P. coatneyi.* Moreover, the continuous detection of these parasites in humans under natural settings suggests their potential to emerge as public health threats, similar to those posed by *P. knowlesi* [15, 16, 44, 45]. Therefore, the assays developed in this study may serve as molecular tools to broaden the current focus of malaria surveillance beyond *P. knowlesi*.

While the present study only demonstrated the application of the developed assays on macaque reservoirs and mosquito vectors, human infections with simian *Plasmodium* species other than *P. knowlesi* have been continuously reported in regions where zoonotic malaria transmission occurs [15, 16, 45, 46]. In such settings, these assays may be implemented to complement conventional clinical diagnostic methods to confirm suspected non-*P. knowlesi* simian malaria infections in humans. This is particularly important in cases where timely and accurate diagnosis is essential for appropriate clinical management, and where conventional methods may lack sufficient sensitivity or specificity. However, further validation of the developed assays using clinical samples would be required before routine diagnostic implementation.

Despite the lower cost and comparable sensitivity of conventional nested PCR assays, the qPCR assays developed in this study offer several important advantages. The most notable advantage of qPCR assay is its ability to rapidly detect the target sequences without the need for post-PCR amplification processing such as gel electrophoresis, thereby reducing turnaround time. Moreover, while genus-level PCR followed by sequencing enables the detection of a wide range of *Plasmodium* species, recent studies have reported difficulties in successfully sequencing the *Plasmodium* 18S rRNA gene from several *Plasmodium*-positive *Anopheles* samples identified by genus-specific PCR assays, often due to the very low copy number of parasite DNA [28, 29]. In contrast, the qPCR assays developed in this study allow sensitive detection of *Plasmodium* DNA without reliance on downstream sequencing, making them suitable for screening mosquito samples with low parasite loads.

Furthermore, unlike nested PCR which requires sequential amplification steps, qPCR is performed in a single closed-tube reaction which is less labour-intensive and helps to minimise the risk of carryover contamination [39, 47]. In addition, qPCR allows for the real-time detection and quantification of parasite DNA, thereby providing information on parasite burden that cannot be obtained from nested PCR assays [30]. These features highlight the relevance of the developed assays as complementary diagnostic tools for targeted surveillance and improved characterisation of zoonotic malaria transmission, particularly in endemic regions such as Malaysian Borneo.

The slightly lower amplification efficiency observed in the Pcyprobe1 assay may be associated with intra- and intermolecular interactions among the primers and probes, potentially resulting in secondary structure formation that interferes with amplification [48]. Despite this, the amplification efficiencies obtained for the developed assays were comparable to that reported for the *P. knowlesi*-specific qPCR assay (E = 95%) described by Divis et al. [31].

To date, no published studies have reported the use of probe-based qPCR assays for the detection of *P. inui* and *P. coatneyi*. Although a TaqMan qPCR assay for *P. cynomolgi* was recently described, the oligonucleotide sequence of the *P. cynomolgi*-specific probe reported in that study differs from the probe developed in the present study [49]. The previous study utilised PCR primers specific to *P. cynomolgi*, whereas the present study employed a single set of *Plasmodium* genus-specific primers to amplify the conserved SSU rRNA region across all *Plasmodium* species. The incorporation of species-specific PCR primers may limit assay performance, as sequence variation among different *P. cynomolgi* genotypes could result in primer-template mismatches, potentially leading to false-negative results [50, 51].

In recent years, several studies have described the development of probe-based multiplex qPCR assays for the simultaneous detection and quantification of multiple *Plasmodium* species [32, 49, 52]. In contrast, the present study utilised singleplex qPCR assays to allow independent detection and quantification of each target *Plasmodium* species. Although multiplex assays are generally faster and less labour-intensive, their design and optimisation are considerably more challenging [53, 54]. Furthermore, amplification efficiency and assay specificity may be compromised due to competition among multiple primers and probes present within the same reaction mixture [53].

Overall, the development of species-specific qPCR assays in the present study provides valuable tools for advancing research on zoonotic malaria. Although *P. knowlesi* is currently recognised as the primary cause of zoonotic malaria in Southeast Asia, growing evidence indicates that other simian malaria parasites, such as *P. cynomolgi*, are also capable of infecting humans [15, 16, 55]. Consequently, the availability of sensitive and specific molecular diagnostic methods is crucial for identifying emerging infections and assessing the public health importance of these parasites.

## Conclusion

Molecular-based assays to accurately identify *P. inui, P. cynomolgi* and *P. coatneyi* were successfully developed and optimised in this present study. The assays were demonstrated to be highly species-specific and were able to detect at least five parasites. Moreover, they are more rapid and less laborious than the conventional nested PCR assays, making them suitable for routine diagnostic testing. Given the limitations of microscopy technique, the assays could serve as a more useful tool to detect and distinguish between a wide variety of morphologically similar *Plasmodium* parasites. The assays are also suitable for malaria infection with very low parasite burden, particularly among asymptomatic patients, as well as in mosquito or faecal samples.

## Supplementary Information

Additional file 1. Extracted plasmid DNA from *E. coli* glycerol stocks carrying recombinant plasmids with 18S rRNA gene inserts of *P. inui*, *P. coatneyi* and *P. cynomolgi*

Additional file 2. Referral 18S rRNA gene sequences of different *Plasmodium* species used in the sequence alignment

Additional file 3. Genomic DNA samples of human *Plasmodium* species tested for specificity using newly-developed qPCR assays for *P. inui, P. coatneyi*, and *P. cynomolgi*

Additional file 4. Detection of *P. inui, P. coatneyi* and *P. cynomolgi* A-type SSU rRNA genes in serially diluted plasmid DNA by qPCR assays

## Data Availability

All data generated or analysed throughout this study are included within this published article and its additional files.
