# Supplementary material for: Accurate and sensitive TaqMan real-time PCR assays to detect and quantify simian malaria parasites Plasmodium cynomolgi, Plasmodium inui and Plasmodium coatneyi
Source: Malar J. 2026 Jun 8;25:294. doi: 10.1186/s12936-026-05955-4 (PMC13465652; doi:10.1186/s12936-026-05955-4)
Supplement: Supplementary file 1 [file 12936_2026_5955_MOESM1_ESM.docx]

**Additional File 1**: Extracted plasmid DNA from *E. coli* glycerol stocks carrying recombinant plasmids with 18S rRNA gene inserts of *P. inui*, *P. coatneyi* and *P. cynomolgi*

| ***Plasmodium* species**  **(A-type 18S rRNA insert)** | **Date prepared for glycerol stocks** | **Plasmid DNA concentration**  **(ng/μL)** | **A_260/280_** | **A_260/230_** |
| --- | --- | --- | --- | --- |
| *P. inui* | 24/04/2005 | 85.34±2.02 | 1.84 ±0.02 | 2.04±0.04 |
| *P. coatneyi* | 12/05/2005 | 77.45±1.73 | 1.88±0.02 | 1.91±0.02 |
| *P. cynomolgi* | 23/02/2006 | 70.31±1.20 | 1.82±0.04 | 2.02±0.02 |

The glycerol stocks of *E. coli* transformant with plasmid DNA containing the partial A-type 18S rRNA gene inserts were prepared and used in previous study (20). “Date prepared” refers to the original preparation date of the glycerol stocks, stored and maintained at -80 °C. Each measurement was performed in triplicate to ensure accuracy and minimise experimental error. The values of concentration and absorbance ratios are presented as mean ± standard error of the mean. The concentrations of the extracted plasmid DNA ranged from 70.31-85.34 ng/μL, while their purity were within acceptable ranges.
