# Supplementary material for: Accurate and sensitive TaqMan real-time PCR assays to detect and quantify simian malaria parasites Plasmodium cynomolgi, Plasmodium inui and Plasmodium coatneyi
Source: Malar J. 2026 Jun 8;25:294. doi: 10.1186/s12936-026-05955-4 (PMC13465652; doi:10.1186/s12936-026-05955-4)
Supplement: Supplementary file 2 [file 12936_2026_5955_MOESM2_ESM.docx]

**Additional File 2 :** Referral 18S rRNA gene sequences of different *Plasmodium* species used in the sequence alignment.

| ***Plasmodium* species** | **Accession number** | **SSU rRNA gene isoform** | **Host** | **Reference** |
| --- | --- | --- | --- | --- |
| *P. inui* | AB287276 | A-type | *Macaca cyclopis* | Nishimoto, Arisue (48) |
|  | AB287277 |  | *Macaca fascicularis* |  |
|  | FJ619072 |  |  | Divis et al., Unpublished |
|  | FJ619078 |  |  |  |
|  | FJ619093 |  |  |  |
|  | MN368105 |  | *An. balabacensis* | Ang, Kadir (28) |
|  | FJ619072 | S-type | *Macaca fascicularis* | Divis et al., Unpublished |
|  | AB287275 |  | *Cynopithecus niger* | Nishimoto, Arisue (48) |
| *P. cynomolgi* | AB287289 | A-type | *Macaca nemestrina* | Nishimoto, Arisue (48) |
|  | AB287290 |  | *Macaca radiata* |  |
|  | FJ619084 |  | *Macaca fascicularis* | Divis et al., Unpublished |
|  | L07559 |  | *Rhesus monkey* | Waters, Higgins (49) |
|  | MN368117 |  | *An. balabacensis* | Ang, Kadir (28) |
|  | AB287288 | S-type | *Macaca fascicularis* | Nishimoto, Arisue (48) |
|  | L08242 |  | *Macaca mulatta* | Corredor and Enea (1994) |
| *P. coatneyi* | AB265790 | A-type | *Macaca fascicularis* | Nishimoto, Arisue (48) |
|  | AB265791 |  |  |  |
|  | FJ619092 |  |  | Divis et al., Unpublished |
|  | FJ619094 |  |  |  |
|  | MN535353 |  | *An. roperi* | Ang et al., Unpublished |
|  | AB265789 | S-type | *Macaca fascicularis* | Nishimoto, Arisue (48) |
| *P. knowlesi* | DQ641521 | A-type | *Macaca fascicularis* | Lee et al., Unpublished |
|  | FJ619069 |  |  | Divis et al., Unpublished |
|  | AY327550 |  | Human | Singh, Kim Sung (8) |
|  | AY327557 |  |  |  |
|  | DQ350262 | S-type | Human | Lee et al., Unpublished |
| *P. fieldi* | FJ619063 | A-type | *Macaca fascicularis* | Divis et al., Unpublished |
|  | FJ619101 |  |  |  |
|  | FJ619102 | S-type |  |  |
| *P. simiovale* | AB287286 | A-type | *Macaca sinica* | Nishimoto, Arisue (48) |
|  | AB287285 | S-type |  |  |
| *P. falciparum* | M19172 | A-type | Human | McCutchan, de la Cruz (50) |
| *P. vivax* | U07367 | A-type | Mosquito | Li, Wirtz (51) |
|  | U03079 |  | Human |  |
|  | U07368 | S-type | Mosquito |  |
|  | U03080 |  | Human | Qari, Goldman (52) |
| *P. malariae* | KX672018 | Not specified | Human | Li, Zhao (53) |
|  | KX672020 | Not specified |  |  |
|  | M54897 | A-type |  | Goman, Mons (54) |
| *P. ovale* | L48986 | A-type | Human | Qari, Shi (55) |
