# Supplementary material for: Accurate and sensitive TaqMan real-time PCR assays to detect and quantify simian malaria parasites Plasmodium cynomolgi, Plasmodium inui and Plasmodium coatneyi
Source: Malar J. 2026 Jun 8;25:294. doi: 10.1186/s12936-026-05955-4 (PMC13465652; doi:10.1186/s12936-026-05955-4)
Supplement: Supplementary file 3 [file 12936_2026_5955_MOESM3_ESM.docx]

**Additional File 3:** Genomic DNA samples of human *Plasmodium* species tested for specificity using newly-developed qPCR assays for *P. inui, P. coatneyi*, and *P. cynomolgi*.

| ***Plasmodium* species** | **Sample ID** | **Parasitaemia (parasites / µL blood)** | **Travel history** | **Sample collection date (Month/Year)** | **References** |
| --- | --- | --- | --- | --- | --- |
| *P. falciparum* | KG070 | 32,600 | Worked in Africa | December 2018 | Yunos et al., 2022 |
|  | KG083 | 33,396 | Worked in Africa | January 2019 | Yunos et al., 2022 |
|  | KG120 | 6,956 | Worked in Africa | April 2019 | Yunos et al., 2022 |
| *P. vivax* | KG059 | 8,208 | Worked in Papua New Guinea | December 2018 | Yunos et al., 2022 |
|  | KG154 | 6,200 | Worked in Papua New Guinea | May 2019 | Yunos et al., 2022 |
|  | KG127 | 10,080 | Worked in Papua New Guinea | April 2019 | Yunos et al., 2022 |
| *P. ovale* | KG063 | 116,000 | Worked in Papua New Guinea | November 2018 | Yunos et al., 2022 |
|  | KG192 | 1,268 | Worked in Africa | August 2019 | Yunos et al., 2022 |
|  | KG263 | 5,520 | Worked in Africa | October 2019 | Yunos et al., 2022 |
| *P. malariae* | KG041 | 7,120 | Worked in Africa | December 2018 | Yunos et al., 2022 |
|  | KT177 | 4,633 | Worked in Africa | May 2014 | Hu et al., 2021 |
|  | KT353 | 2,928 | Worked in Africa | May 2015 | Hu et al., 2021 |

**Note**: As indicated in previous studies, all genomic DNA was extracted from whole blood samples obtained from patients admitted to Kapit Hospital, Sarawak. These samples were confirmed as single *Plasmodium* species infections by nested PCR assays. As there were no indigenous human *Plasmodium* species infections in Sarawak, all patients had a travel history to malaria-endemic countries. In the present study, none of the samples showed amplifications when tested with the newly developed qPCR assays for *P. inui, P. coatneyi,* and *P. cynomolgi*.

**References:**

1. Hu, T. H., N. Rosli, D. S. A. Mohamad, K. A. Kadir, Z. H. Ching, Y. H. Chai, N. N. Ideris, L. S. C. Ting, A. A. Dihom, S. L. Kong, E. K. Y. Wong, J. E. H. Sia, T. Ti, I. P. F. Chai, W. Y. Tang, K. C. Hii, P. C. S. Divis, T. M. E. Davis, C. Daneshvar and B. Singh (2021). "A comparison of the clinical, laboratory and epidemiological features of two divergent subpopulations of Plasmodium knowlesi." Sci Rep **11**(1): 20117.
2. Yunos, N. E., H. M. Sharkawi, K. C. Hii, T. H. Hu, D. S. A. Mohamad, N. Rosli, T. Masron, B. Singh and P. C. S. Divis (2022). "Spatio-temporal distribution and hotspots of Plasmodium knowlesi infections in Sarawak, Malaysian Borneo." Scientific Reports **12**(1): 17284.
