# Supplementary material for: Accurate and sensitive TaqMan real-time PCR assays to detect and quantify simian malaria parasites Plasmodium cynomolgi, Plasmodium inui and Plasmodium coatneyi
Source: Malar J. 2026 Jun 8;25:294. doi: 10.1186/s12936-026-05955-4 (PMC13465652; doi:10.1186/s12936-026-05955-4)
Supplement: Supplementary file 4 [file 12936_2026_5955_MOESM4_ESM.docx]

| ***Plasmodium* species** | **Plasmid DNA concentration (copies/μL)** | **qPCR assays** | | |
| --- | --- | --- | --- | --- |
|  |  | ***P. inui*** | ***P. coatneyi*** | ***P. cynomolgi*** |
|  |  | **C*t*** | **C*t*** | **C*t*** |
| *P. inui* | 1 × 10^6^ | 25.0 | n.d. | n.d. |
|  | 1 × 10^5^ | 27.7 |  |  |
|  | 1 × 10^4^ | 31.4 |  |  |
|  | 1 × 10^3^ | 35.2 |  |  |
|  | 1 × 10^2^ | 38.7 |  |  |
|  | 10 | 41.4 |  |  |
|  | 1 | ND |  |  |
| *P. coatneyi* | 1 × 10^6^ | n.d. | 26.5 | n.d. |
|  | 1 × 10^5^ |  | 28.3 |  |
|  | 1 × 10^4^ |  | 32.0 |  |
|  | 1 × 10^3^ |  | 35.6 |  |
|  | 1 × 10^2^ |  | 39.0 |  |
|  | 10 |  | 43.2 |  |
|  | 1 |  | ND |  |
| *P. cynomolgi* | 1 × 10^6^ | n.d. | n.d. | 26.1 |
|  | 1 × 10^5^ |  |  | 29.7 |
|  | 1 × 10^4^ |  |  | 34.4 |
|  | 1 × 10^3^ |  |  | 37.2 |
|  | 1 × 10^2^ |  |  | 39.9 |
|  | 10 |  |  | 43.6 |
|  | 1 |  |  | ND |

**Additional File 4:** Detection of *P. inui, P. coatneyi* and *P. cynomolgi* A-type SSU rRNA genes in serially diluted plasmid DNA by qPCR assays,

C*t***:** Cycle threshold value. Each value corresponds to amplification of the target 18S rRNA gene in the 10-fold serially diluted plasmid DNA. Lower values indicate higher initial template concentration. The lowest detectable concentration for all assays was 10 copies per reaction, indicating the limit of detection. n.d.: no amplification detected.
